# Prenatal n–3 LC-PUFA intake and the incidence of allergic diseases in offspring: an updated systematic review and meta-analysis

**DOI:** 10.3389/fnut.2026.1923649

**Published:** 2026-09-24

**Authors:** Tingwen Zheng, Shoumeng Yan, Tianci Fan, Yue Zhai, Yuxue Jiang, WenHui Xu, Huimin Tian

**Affiliations:** 1 School of Nursing, Jilin University, Changchun, Jilin, China; 2 China-Japan Union Hospital of Jilin University, Changchun, Jilin, China; 3 School of Public Health, Jilin University, Changchun, Jilin, China

**Keywords:** allergic diseases, meta-analysis, n–3 long-chain polyunsaturated fatty acid, offspring, pregnancy

## Abstract

**Background:**

Allergic diseases are among the most prevalent conditions affecting children’s health worldwide. Maternal intake of n–3 long-chain polyunsaturated fatty acid (n–3 LC-PUFA) during pregnancy has been suggested as a strategy to reduce allergic disease risk in offspring; however, existing evidence remains inconsistent. This systematic review and meta-analysis aims to evaluate the association between prenatal n–3 LC-PUFA intake and the risk of allergic diseases in offspring.

**Methods:**

A comprehensive search of 11 databases was conducted from inception to December 12, 2025. Prospective cohort studies and RCTs investigating prenatal n–3 LC-PUFA intake and allergic diseases in offspring were included. Data synthesis was performed using RevMan 5.4 and Stata 18.0. Heterogeneity, sensitivity, subgroup, and publication bias analyses were conducted. The certainty of evidence was assessed using the GRADE approach.

**Results:**

Eight prospective cohort studies and eight RCTs were included. Cohort studies indicated that prenatal n–3 LC-PUFA intake was significantly associated with a lower risk of overall allergic diseases (RR = 0.88, 95% CI: 0.82–0.95, *p* < 0.001) and asthma (RR = 0.81, 95% CI: 0.68–0.96, *p* = 0.02). RCTs demonstrated a significant reduction in the risk of egg allergy (RR = 0.47, 95% CI: 0.28–0.80, *p* = 0.006) and other specific allergic conditions, with a lower risk observed among European populations (RR = 0.76, 95% CI: 0.60–0.96, *p* = 0.02).

**Conclusion:**

Prenatal n–3 LC-PUFA intake may be associated with a lower risk of allergic diseases in offspring, particularly asthma and egg allergy, and may confer population-specific benefits, especially in European populations. Further high-quality studies are needed to confirm these findings and clarify their implications for prenatal nutritional guidance.

**Systematic review registration:**

https://www.crd.york.ac.uk/PROSPERO/view/CRD420251237192, Identifier: CRD420251237192.

## 1 Introduction

Allergic diseases represent a major global public health challenge in children, with increasing prevalence worldwide (1–4). Atopic dermatitis, IgE-mediated food allergies, allergic asthma, and rhinoconjunctivitis are among the most common chronic immune disorders in childhood, posing major challenges for family care and pediatric public health management. The occurrence of allergic diseases is closely related to fetal immune development during the prenatal period (5–8). The gestational period, especially the second and third trimesters, is a critical window for immune system differentiation and the establishment of immune tolerance (9). During this period, maternal nutritional exposures can directly influence the trajectory of fetal immune development through the placenta. Consequently, dietary and nutritional interventions during pregnancy have become an increasingly important strategy for preventing allergic diseases in offspring. Compared with postnatal interventions, prenatal interventions offer greater preventive value at the source.

In the primary prevention of allergic diseases, fish intake has received particular attention, especially oily fish rich in n–3 LC-PUFAs (10, 11), such as docosahexaenoic acid (DHA) and eicosapentaenoic acid (EPA). The n–3 LC-PUFAs (EPA and DHA), as key nutrients during pregnancy, can maintain immune balance by regulating the Th1/Th2 ratio and inhibiting the release of pro-inflammatory cytokines (12). This biological mechanism is presumed to be closely related to the development of allergic diseases in offspring.

However, current evidence regarding whether maternal intake of n–3 LC-PUFAs during pregnancy reduces the risk of allergic diseases in offspring remains inconsistent: some studies (13–15) have shown that supplementation with n–3 LC-PUFA during pregnancy can reduce the risk of allergic diseases in offspring; other studies (16–19) have reported no statistically significant reduction in the incidence of allergic diseases, and even some studies (5) have found a negative correlation between n–3 LC-PUFA supplement intake and related allergic outcomes. Several previous systematic reviews and meta-analyses have addressed this topic. One previous study (20) was based on the evidence available up to 2016 and reported significant effects only for eczema, egg allergy, and several sensitization outcomes during infancy, whereas no significant associations were observed for asthma, rhinoconjunctivitis, or allergic diseases beyond 12 months of follow-up. Subsequently, another study (21) reported a reduced risk of egg and peanut allergies but no significant effects on other allergic outcomes (such as eczema, asthma/wheezing, etc.). Previous reviews primarily analyzed allergic diseases in offspring with a follow-up period of 12–36 months, lacking data extending to school age (22).

Despite these advances, several limitations persist in the existing literature. First, the time frames of previous reviews were relatively long, and recently published studies were not included, resulting in outdated evidence. Second, the follow-up periods were relatively short or limited to RCTs (21, 22). Since the publication of the most recent systematic review, one additional randomized controlled trial and two large prospective cohort studies have become available, providing new evidence on the association between prenatal n–3 LC-PUFA intake and allergic diseases in offspring. More importantly, previous systematic reviews and meta-analyses did not always account for differences in study design by conducting separate statistical analyses of randomized controlled trials and prospective cohort studies. Therefore, in this study, we analyzed these two study designs separately. Moreover, the included data have not been analyzed by subgroups based on dimensions such as study region, follow-up time, and type of exposure factor, and no distinction has been made in the effects. Therefore, given the substantial burden of allergic diseases and the uncertainty of the current evidence, we conducted an updated systematic review and meta-analysis by incorporating the latest available studies and separately synthesizing evidence from randomized controlled trials and prospective cohort studies to clarify the association between maternal n–3 long-chain polyunsaturated fatty acid (LC-PUFA) intake during pregnancy and allergic diseases in offspring, thereby providing evidence-based support for nutritional guidance during pregnancy and the early prevention of allergic diseases.

## 2 Materials and methods

### 2.1 Protocol and registration

This meta-analysis has been registered in PROSPERO (Registration No. CRD420251237192, https://www.crd.york.ac.uk/PROSPERO/view/CRD420251237192). The review was conducted and reported in accordance with the Preferred Reporting Items for Systematic Reviews and Meta-Analyses (PRISMA) 2020 statement. The completed PRISMA 2020 checklist is provided in Supplementary Table S2.

### 2.2 Search strategy

A structured and comprehensive literature search was conducted from inception to December 12, 2025, using the following databases: PubMed, Web of Science, MEDLINE, CINAHL, Embase, SCOPUS, the Cochrane Library, and four Chinese databases (CNKI, SinoMed, WanFang Data, and the VIP Database). The search strategy was developed using a combination of pregnancy-related terms, n–3 long-chain polyunsaturated fatty acid (n–3 LC-PUFA)-related terms, offspring-related terms, and allergic disease-related terms. The following keywords were used in the literature search: pregnancy, maternal, perinatal, n–3 PUFA, DHA, EPA, fish oil, offspring, infant, child, asthma, atopy, allergic rhinitis, atopic dermatitis, and food allergy. The detailed search strategy is provided in Supplementary material.

### 2.3 Eligibility criteria

The meta-analysis included studies that met the following criteria: (1) The study designs included randomized controlled trials and prospective cohort studies. (2) Eligible participants were pregnant women and their children born after delivery. (3) The intervention/exposure was the intake of n–3 long-chain polyunsaturated fatty acid (LC-PUFA) through diet or supplementation during pregnancy. (4) The outcomes were physician-diagnosed IgE-mediated allergic diseases, including atopic dermatitis (eczema), asthma (wheeze), allergic rhinitis, and food allergies. (5) Studies were excluded if they lacked primary data, were animal studies, cross-sectional studies, case–control studies, reviews, or conference papers. Studies that examined only maternal intake or supplementation of n–3 LC-PUFA during the postpartum period (either through breastfeeding or direct supplementation to infants) were also excluded.

### 2.4 Outcome measures

The primary outcomes of this review were the risks of atopic diseases in offspring during infancy, childhood, or adolescence. These included the risk of any IgE-mediated allergic disease, positive skin prick test (SPT) results, and specific conditions such as atopic dermatitis (eczema), allergic asthma (wheeze), food allergy, and allergic rhinitis. The definitions and assessment methods of allergic outcomes varied across the included studies, including physician diagnosis, parental report, questionnaire-based assessment, and laboratory-based sensitization measures, depending on the outcome and study.

### 2.5 Data extraction

Two authors independently assessed the eligibility of the articles for inclusion. Any disagreements were resolved through discussion, and if necessary, by consultation with a third-party arbitrator. The following data were extracted from the included studies: first author, publication year, country, study design, study population, maternal exposure or supplementation with fish or n–3 long-chain polyunsaturated fatty acids, intervention timing and duration, sample size and number of outcome events, and follow-up duration. The extracted data were entered into a standardized Microsoft Word document.

### 2.6 Risk of bias assessment

This study used standardized tools to assess the risk of bias for the included randomized controlled trials (RCTs) and prospective cohort studies, respectively. For RCTs, the Cochrane Risk of Bias tool (RoB 2.0) (23) was used to evaluate the risk of bias in five domains: the randomization process, deviations from intended interventions, missing outcome data, measurement of the outcome, and selection of the reported results. The risk levels in each domain are classified as “low risk of bias,” “some concerns,” and “high risk of bias.” The results of the risk of bias assessment were visualized using Review Manager (RevMan) software. For prospective cohort studies, the Risk Of Bias In Non-randomized Studies - of Exposures (ROBINS-E) (24) was used for risk-of-bias assessment. The assessment addresses bias within seven domains: (1) Bias due to confounding (2) Bias arising from measurement of the exposure (3) Bias in selection of participants into the study (4) Bias due to post-exposure interventions (5) Bias due to missing data (6) Bias in measurement of the outcome (7) Bias in selection of the reported result. The risk of bias in each domain was graded as either low risk of bias, some concerns, high risk of bias, or very high risk of bias. The risk of bias assessment was independently completed by two researchers. In case of disagreement, consensus was reached through discussion or consultation with a third researcher.

### 2.7 Certainty of evidence

The certainty of evidence for each critical outcome was assessed using the Grading of Recommendations Assessment, Development and Evaluation (GRADE) system (25). Because RCTs and prospective cohort studies provide different levels of initial certainty, the two study designs were evaluated separately. Evidence certainty was rated as high, moderate, low, or very low according to the domains of risk of bias, inconsistency, indirectness, imprecision, and publication bias. Summary of Findings (SoF) tables were generated using GRADEpro GDT. Two of the authors independently evaluated the research. In case of any disagreement, the third author coordinated and resolved the issue.

### 2.8 Data synthesis and analysis

This study utilized Review Manager (RevMan) 5.4 and Stata 18.0 for statistical analysis. The risk ratio (RR) and 95% confidence interval (CI) were used to assess the impact of n–3 long-chain polyunsaturated fatty acid (n–3 LC-PUFA) intake during pregnancy on allergic diseases in offspring. Review Manager 5.4 was used to pool the sample sizes and event counts of the included studies, and a random-effects model was applied to estimate the pooled effect sizes, given the anticipated clinical and methodological heterogeneity across studies. Event-based RRs were used as the primary effect measure because this approach provided a consistent effect measure across the included randomized controlled trials and prospective cohort studies, while event counts were more consistently available across studies than directly reported adjusted effect estimates. Multivariable-adjusted estimates were not used in the primary analysis because the reported effect measures, exposure definitions, reference categories, and adjustment sets varied across studies, limiting their comparability. Heterogeneity was assessed using the I^2^ statistic in conjunction with Cochran’s Q statistic (26). Sensitivity analyses were performed using a fixed-effect model to assess the robustness of the pooled estimates. Sensitivity analyses for randomized controlled trials and prospective cohort studies included leave-one-out analyses, in which each study was sequentially excluded and the meta-analysis repeated, and comparisons between fixed-effects and random-effects models to assess the robustness of the pooled estimates. Additionally, for prospective cohort studies, a sensitivity analysis was performed by excluding studies judged to be at very high risk of bias according to the ROBINS-E tool to assess the robustness of the pooled estimates. Publication bias was assessed using funnel plot inspection and Egger’s test, with a *p* < 0.05 considered indicative of statistically significant funnel plot asymmetry.

### 2.9 Subgroup analysis and its grouping basis

We also conducted subgroup analyses, mainly based on study region, the EPA-to-DHA ratio, follow-up duration, intervention initiation time, disease type, exposure type, and maternal age.

The basis for subgrouping was as follows: To explore the relationship between the risk of allergic diseases and age, follow-up duration was divided into three subgroups—infancy, toddlerhood, and preschool age and above—based on the classic sequence and progression of eczema, food allergy, asthma, and allergic rhinitis described in the ‘allergic march’ theory (27, 28). The rationale for these subgroup classifications is as follows: Studies have shown that atopic dermatitis (eczema) typically emerges in infancy and is often the first manifestation of an allergic process. The first year of life represents the peak period of its occurrence (28, 29). The period from 1 to 3 years of age represents a transitional stage in the atopic process. During this stage, early allergic diseases may either be the critical period of remission or persist, and susceptibility to asthma begins to emerge. This period represents a critical transition from cutaneous and gastrointestinal allergies to respiratory allergic diseases (30). Large-scale follow-up data from the Danish National Birth Cohort confirm that this stage is a critical transitional period for disease outcome. After entering the preschool period, ages 3–5 years represent the core stage of progression in the atopic process, covering the main onset periods of asthma and allergic rhinitis and is a critical period for the onset of respiratory allergy in the atopic process. Some studies have shown (17) that maternal fish oil intake during pregnancy significantly reduces the risk of recurrent wheezing and asthma in children aged 3–5 years. Similarly, a Finnish study reported that reduced maternal intake of n–3 polyunsaturated fatty acids during pregnancy increases the risk of asthma in 5-year-old children (31), which is consistent with the peak incidence data from the ISAAC study (32) and the Danish National Birth Cohort (33); Beyond 5 years of age, the atopic process enters a stable stage, during which the allergic disease phenotype is largely established. Unresolved asthma and allergic rhinitis often persist into adolescence or even adulthood, enabling evaluation of the long-term protective effects of early interventions. This feature has been confirmed in long-term follow-up studies of the Danish National Birth Cohort (33). The categorization of maternal age was based on clinical considerations and the characteristics of the available evidence. Although maternal age ≥35 years is widely recognized as the criterion for advanced maternal age (34), the included studies did not specifically examine populations with advanced maternal age. We therefore did not use 35 years as the cutoff. Previous studies have suggested that maternal age around 30 years may be associated with relatively favorable reproductive and pregnancy outcomes (35–38). Accordingly, we used 30 years as an exploratory cutoff and categorized maternal age as <30 years and ≥30 years. Given the limited number of studies available for this subgroup analysis, the results should be interpreted cautiously. Therefore, maternal age was categorized as <30 years and ≥30 years. The classification of intervention initiation time was based on evidence regarding fetal immune development. Previous studies indicate that the period before 20 weeks of gestation represents a critical window for fetal immune cell differentiation. Supplementation with n–3 polyunsaturated fatty acids is recommended to begin in the second trimester, preferably before 20 weeks of gestation, and continue until delivery or about 37 weeks (39, 40). Accordingly, the intervention initiation time was categorized into two subgroups: <20 weeks and ≥20 weeks of gestation.

## 3 Results

### 3.1 Literature screening and characteristics of the included studies

A total of 4,265 records were identified through the database searches, and the study selection process is presented in the PRISMA 2020 flow diagram (Figure 1). Ultimately, eight randomized controlled trials (RCTs) with a combined sample size of 4,271 participants were included, representing populations from Europe, Oceania, and North America. The overall risk of bias across the included RCTs was relatively low. Additionally, eight prospective cohort studies (*n* = 123,445) were included. These studies were conducted in Europe, Asia, Africa, and the Americas. According to the ROBINS-E assessment, three cohort studies were judged to have some concerns, four were at high risk of bias, and one was at very high risk of bias. The main sources of bias were related to participant selection and missing data, whereas one study was judged to be at very high risk of bias because of very high risk of bias in the domains of exposure classification and missing data. The basic characteristics of the included studies are presented in Tables 1, 2, and the quality assessments are provided in Supplementary Figures S1, S2 and Supplementary Table S1. The certainty of evidence varied across outcomes and study designs. For RCTs, the certainty ranged from low to high, whereas the certainty of evidence from prospective cohort studies ranged from very low to low (Table 3).

**Figure 1:**
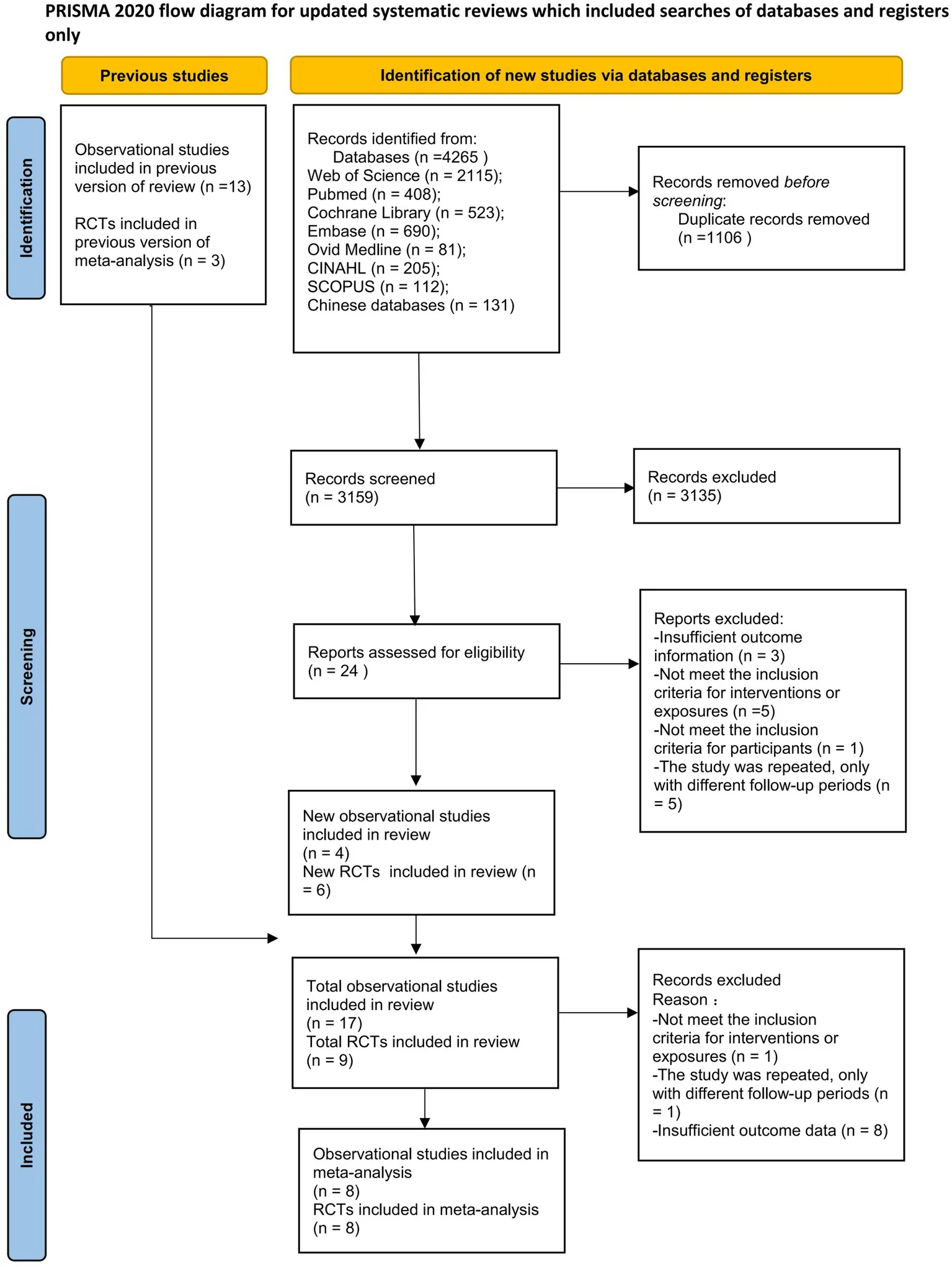
Flow diagram of the literature search process.

**Table 1:** Characteristics of the included randomized controlled trials.

Author	Year	Country	Type of intervention	Outcome	Intervention (events/total)	Control (events/total)	Follow-up
Komulainen et al. (49)	2023	Finland	Fish oil capsule	Any IgE-associated disease	11/65	19/64	24 months
Hansen et al. (50)	2017	Denmark	Fish oil capsule	IgE-associated asthma (EPA-rich)	31/262	28/134	24 months
				IgE-mediated rhinoconjunctivitis	57/262	40/134	
Bisgaard et al. (17)	2016	Denmark	Fish oil capsule	IgE-associated asthma	58/346	78/349	3–5 years
				IgE-associated eczema	114/346	99/349	
				IgE-mediated rhinoconjunctivitis	33/324	45/321	
Berman et al. (5)	2016	USA	Fish oil capsule	IgE-associated asthma (EPA-rich)	13/31	3/27	36 months
				IgE-mediated food reaction (EPA-rich)	4/30	3/27	
				IgE-associated eczema (EPA-rich)	9/31	7/27	
				IgE-associated asthma (EPA-rich)	10/26	3/27	
				IgE-mediated food reaction (DHA-rich)	5/26	3/27	
				IgE-associated eczema (DHA-rich)	4/26	7/27	
Best et al. (16)	2016	Australia	Fish oil capsule	Any IgE-associated disease	116/367	106/336	6 years
Noakes et al. (19)	2012	UK	Salmon	Any positive SPT	6/48	5/38	6 months
Furuhjelm et al. (14)	2011	Sweden	Fish oil capsule	Any IgE-associated disease	6/54	19/62	24 months
Dunstan et al. (51)	2003	Australia	Fish oil capsule	Any positive SPT	9/35	14/37	12 months

EPA, Eicosapentaenoic Acid; DHA, Docosahexaenoic Acid; SPT, Skin Prick Test; IgE, Immunoglobulin E.

**Table 2:** Characteristics of the included prospective cohort studies.

Author	Year	Country	Type of exposure	Outcome	Exposed (events/total)	Non-exposed (events/total)	Follow-up
Henry et al. (52)	2025	Seychelles	Dietary intake	IgE-associated asthma	26/297	22/184	7 years
Tsuji et al. (15)	2024	Japanese	Dietary intake and Supplement intake	IgE-associated asthma	1651/14552	1619/13867	3 years
				IgE-associated eczema	1612/14552	1623/13867	
				IgE-mediated rhinoconjunctivitis	1210/14552	1312/13867	
				IgE-mediated food reaction	1875/14552	1797/13867	
Maslova et al. (18)	2013	Denmark	Dietary intake and Supplement intake	IgE-associated asthma	371/1333	513/213	7 years
Miyake et al. (53)	2013	Japanese	Dietary intake	IgE-associated asthma (EPA + DHA)	77/338	100/338	23–29 months
				IgE-associated eczema (EPA + DHA)	64/338	48/338	
				IgE-associated asthma (fish)	78/338	95/338	
				IgE-associated eczema (fish)	68/338	55/338	
Jedrychowski et al. (54)	2011	Poland, USA	Dietary intake	IgE-associated eczema	38/125	76/176	12 months
Miyake et al. (55)	2009	Japanese	Dietary intake	IgE-associated eczema (EPA)	34/191	39/191	16–24 months
				IgE-associated asthma (EPA)	44/191	48/191	
				IgE-associated eczema (DHA)	30/191	40/191	
				IgE-associated asthma (DHA)	35/191	55/191	
Chatzi et al. (13)	2008	Spain	Dietary intake	Any positive SPT	36/217	34/194	6.5 years
Sausenthaler et al. (56)	2007	Germany	Dietary intake	IgE-associated eczema	122/771	322/1727	2 years

EPA, Eicosapentaenoic Acid; DHA, Docosahexaenoic Acid; SPT, Skin Prick Test; IgE, Immunoglobulin E.

**Table 3:** GRADE assessment for the certainty of evidence.

Study design	Outcome	No. of participants (studies)	Certainty of evidence (GRADE)	Relative effect RR (95% CI)	Risk with placebo/reference
Prospective cohort studies	Atopic diseases in offspring	123,445 (8 studies)	Very lowᵃ	0.88 (0.82–0.95)	124 per 1,000
	Asthma	33,205 (6 studies)	Low	0.81 (0.68–0.96)	137 per 1,000
	Eczema	33,334 (5 studies)	Very lowᵇ	0.93 (0.82–1.07)	131 per 1,000
Randomized controlled trials	Atopic diseases in offspring	4,271 (8 RCTs)	Lowᵃ^,^ᵇ	0.87 (0.72–1.06)	241 per 1,000
	Asthma	2,241 (7 RCTs)	High	0.80 (0.61–1.05)	170 per 1,000
	Eczema	1,929 (7 RCTs)	Lowᶜ^,^ᵈ	1.21 (0.87–1.67)	197 per 1,000
	Food allergy	444 (4 RCTs)	Moderateᵉ	0.75 (0.40–1.41)	142 per 1,000
	Rhinoconjunctivitis	1,863 (4 RCTs)	High	0.83 (0.66–1.03)	147 per 1,000

CI, confidence interval; GRADE, Grading of Recommendations Assessment, Development and Evaluation; LC-PUFAs, long-chain polyunsaturated fatty acids; RR, risk ratio; RCT, randomized controlled trial. The absolute risk difference was calculated from the assumed risk in the placebo/reference group and the relative effect estimate. Negative values indicate fewer events with n–3 LC-PUFAs, whereas positive values indicate more events. ᵃStudies with a relatively large sample size were considered to have a higher risk of bias. ᵇThe 95% CI crosses the null value (RR = 1), and the confidence interval was considered insufficiently precise for clinical interpretation. ᶜModerate heterogeneity was observed (I^2^ = 53%, p = 0.04), with widely varying point estimates (RR 0.39–3.77) and some non-overlapping confidence intervals. ᵈThe confidence interval crosses the null value and is wide (0.87–1.67). ᵉThe funnel plot was clearly asymmetric. GRADE certainty was assessed separately for prospective cohort studies and RCTs because of differences in study design and risk of bias.

### 3.2 Meta-analysis results of the randomized controlled trial studies

Overall, the RCTs showed no significant association between maternal intake of n–3 LC-PUFA supplements during pregnancy and allergic diseases in offspring. (RR = 0.87, 95% CI: 0.72–1.06, *p* = 0.17) (Figure 2). However, subgroup analyses showed a statistically significant lower risk of allergic diseases among offspring in studies conducted in Europe (RR = 0.76, 95% CI: 0.60–0.96, *p* = 0.02); additionally, maternal supplementation of n–3 LC-PUFAs during pregnancy significantly reduced the risk of egg allergy (RR = 0.47, 95% CI: 0.28–0.80, *p* = 0.006); and significant effects were observed when the intervention began during the mid-to-late stages of pregnancy (≥20 weeks of gestation) (Table 4). The formal test for subgroup differences did not show a statistically significant difference across regions (P for interaction = 0.06).

**Figure 2:**
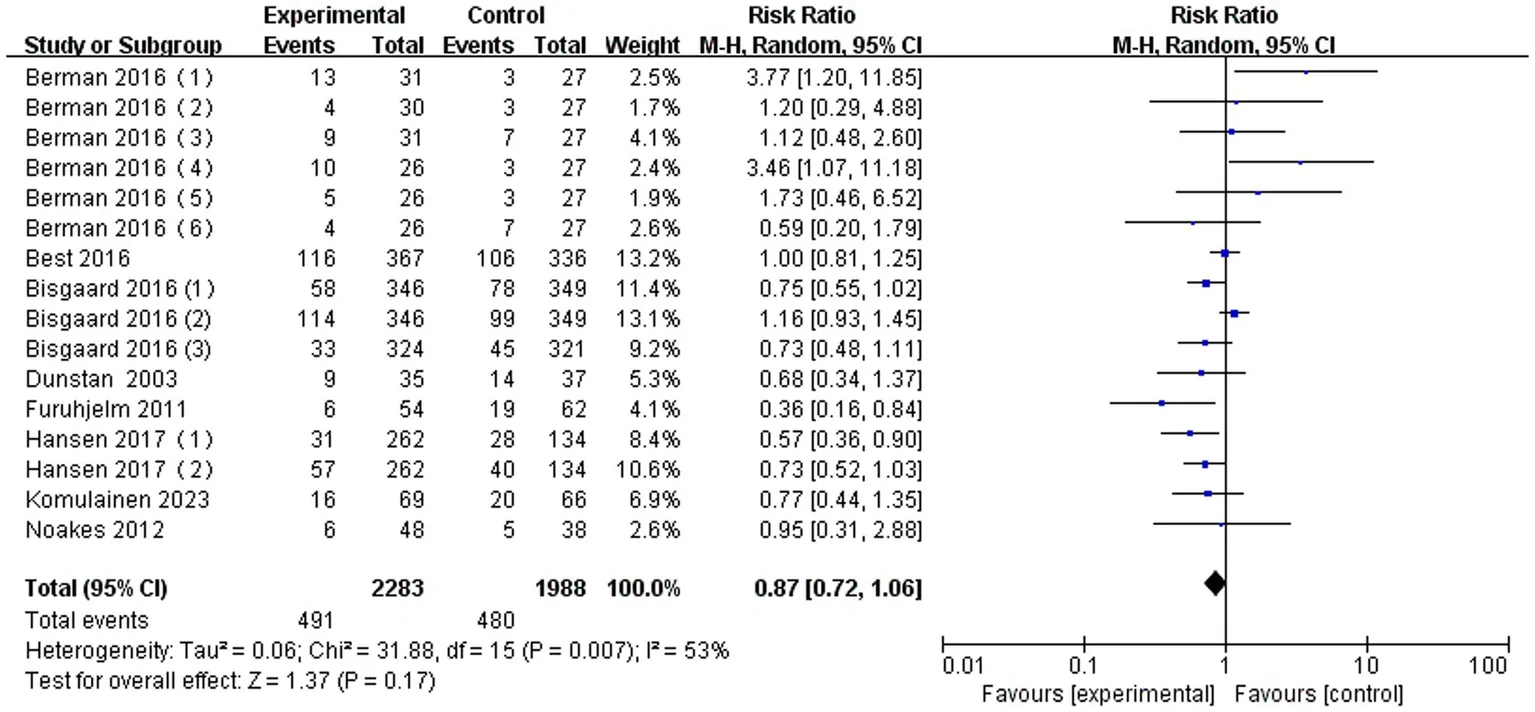
Forest plot of the randomized controlled trials.

**Table 4:** Subgroup analysis of the randomized controlled trials

Subgroup	Number of studies (n)	RR (95% CI)	*I*^2^ (%)	P _overall effect_	P _interaction_
Region		0.87 (0.72, 1.06)	53	0.17	0.06
Oceania	2	0.96 (0.75, 1.22)	8	0.72	
Europe	5	0.76 (0.60, 0.96)	57	0.02	
North America	1	1.58 (0.88, 2.82)	36	0.13	
EPA/DHA		0.87 (0.72, 1.06)	53	0.17	0.48
EPA/DHA ≥ 1	4	0.83 (0.63, 1.09)	66	0.19	
EPA/DHA < 1	5	0.96 (0.72, 1.28)	23	0.77	
Follow-up duration		0.87 (0.72, 1.06)	53	0.17	0.36
Infancy	2	0.75 (0.47, 1.35)	0	0.33	
Toddlerhood	2	0.56 (0.27, 1.17)	53	0.12	
Preschool age and above	4	0.93 (0.75, 1.16)	58	0.54	
Intervention initiation time		0.87 (0.72, 1.06)	53	0.17	0.08
<20 weeks	2	1.33 (0.78, 2.27)	48	0.29	
≥20 weeks	6	0.80 (0.65, 0.98)	57	0.03	
Disease type		0.92 (0.77, 1.10)	43	0.36	0.20
Asthma	7	0.80 (0.61, 1.05)	28	0.11	
Eczema	7	1.21 (0.87, 1.67)	53	0.26	
Food allergy	4	0.75 (0.40, 1.41)	23	0.37	
Rhinoconjunctivitis	4	0.83 (0.66, 1.03)	0	0.09	
Types of food allergies		0.47 (0.30, 0.76)	0	0.002	0.98
Egg allergy	3	0.47 (0.28, 0.80)	0	0.006	
Cow’s milk allergy	2	0.43 (0.11, 1.79)	0	0.25	
Peanut allergy	1	0.53 (0.14, 1.95)	–	0.34	

Sensitivity analyses, conducted by sequentially excluding individual studies and by adjusting the effect model, demonstrated that the pooled effect sizes and 95% confidence intervals remained unchanged, indicating that the results were stable and robust (Supplementary Figures S14, S16). Visual inspection of the funnel plots did not reveal obvious asymmetry, and Egger’s tests did not detect statistically significant asymmetry (*p* > 0.05) (Supplementary Figures S18, S20). According to the GRADE assessment, the certainty of evidence was high for asthma and rhinoconjunctivitis, moderate for food allergy, and low for overall atopic diseases and eczema (Table 3).

### 3.3 Meta-analysis results of the prospective cohort studies

The pooled data from the prospective cohort studies showed that the risk of allergic diseases in the offspring of the exposed group was significantly lower than that of the non-exposed group (RR = 0.88, 95% CI: 0.82–0.95, *p* < 0.001), and the overall association was statistically significant (Figure 3). Subgroup analyses indicated that follow-up duration, exposure type, and disease type were important sources of heterogeneity. Analysis of the asthma subgroup indicated that maternal exposure to n–3 LC-PUFAs was significantly associated with a lower risk of asthma in the offspring (RR = 0.81, 95% CI: 0.68–0.96, *p* = 0.02). Regionally, statistically significant inverse associations were observed in Europe (RR = 0.78, 95% CI: 0.63–0.96, *p* = 0.02) and Asia (RR = 0.93, 95% CI: 0.88–0.99, *p* = 0.03), with higher maternal n–3 LC-PUFA exposure being associated with a lower risk of allergic diseases among offspring in these regions. The formal test for subgroup differences did not show a statistically significant difference across regions (P for interaction = 0.11). Additionally, exposure to n–3 LC-PUFAs during pregnancy was significantly associated with a lower risk of allergic diseases in offspring during infancy (RR = 0.70, 95% CI: 0.51–0.96, *p* = 0.03) and early childhood (Toddlerhood: RR = 0.93, 95% CI: 0.88–0.98, *p* = 0.01; Preschool age and above: RR = 0.71, 95% CI: 0.60–0.85, *p* < 0.001) (Table 5).

**Figure 3:**
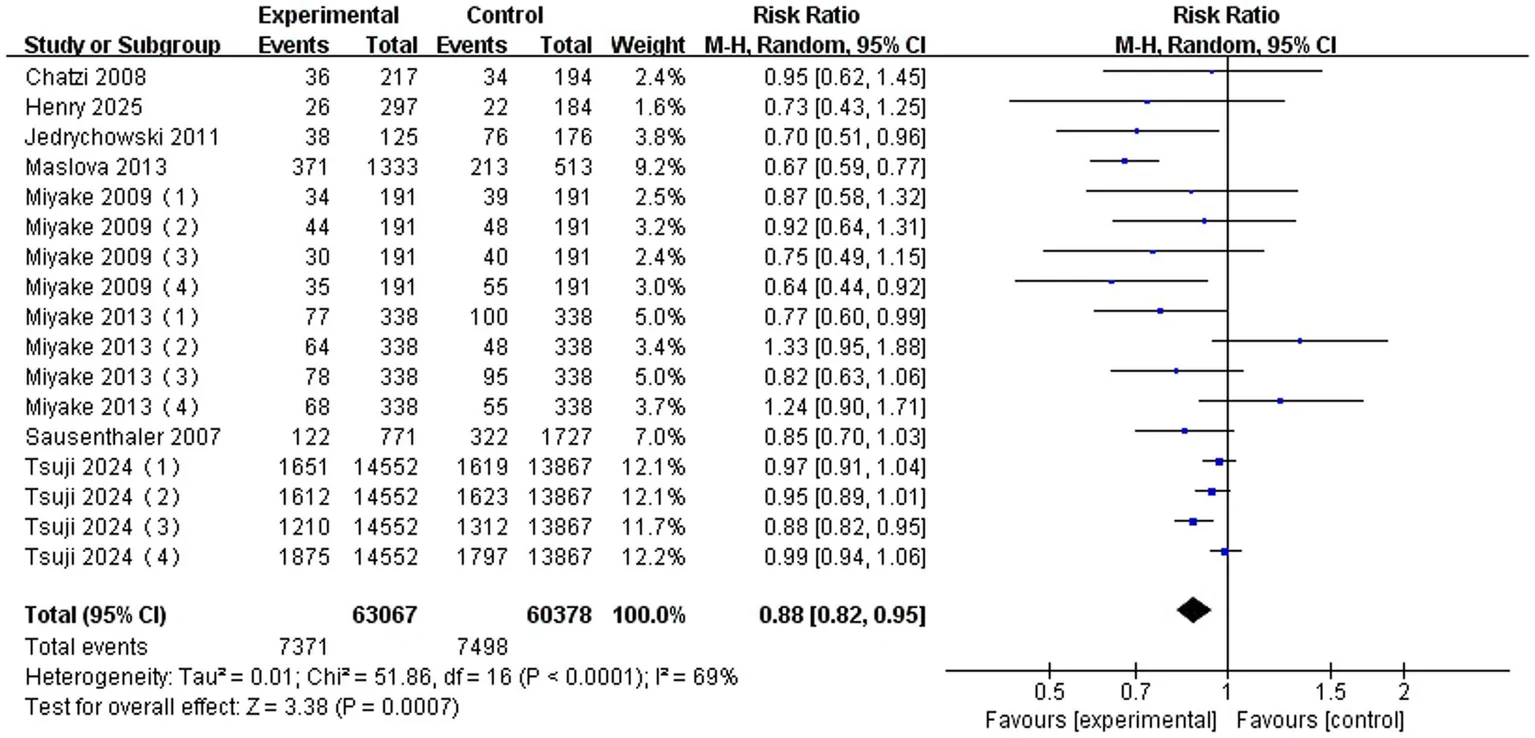
Forest plot of the prospective cohort studies.

**Table 5:** Subgroup analysis of the prospective cohort studies.

Subgroup	Number of studies (n)	RR (95% CI)	*I*^2^ (%)	P _overall effect_	P _interaction_
Region		0.88 (0.82, 0.95)	69	0.0007	0.11
Europe	3	0.78 (0.63, 0.96)	64	0.02	
Asia	3	0.93 (0.88, 0.99)	52	0.03	
Africa	1	0.73 (0.43, 1.25)	–	0.26	
Across continents	1	0.70 (0.51, 0.95)	–	0.03	
Follow-up duration		0.88 (0.82, 0.95)	69	0.0007	0.006
Infancy	1	0.70 (0.51, 0.96)	–	0.03	
Toddlerhood	4	0.93 (0.88, 0.98)	50	0.01	
Preschool age and above	3	0.71 (0.60, 0.85)	15	0.0001	
Exposure type		0.88 (0.82, 0.95)	69	0.0007	<0.001
Dietary intake	6	0.75 (0.68, 0.82)	2	<0.001	
Dietary intake and supplement intake	2	0.95 (0.89, 1.01)	59	0.10	
Maternal age (years)		0.86 (0.74, 1.00)	47	0.05	0.16
<30 yr	2	0.71 (0.54, 0.93)	0	0.01	
≥30 yr	2	0.89 (0.75, 1.06)	52	0.20	
Disease type		0.88 (0.82, 0.95)	70	0.001	0.03
Asthma	6	0.81 (0.68, 0.96)	78	0.02	
Eczema	5	0.93 (0.82, 1.07)	52	0.31	
Food allergy	1	0.99 (0.94, 1.06)	–	0.85	
Rhinoconjunctivitis	1	0.88 (0.82, 0.95)	–	0.0007	

Sensitivity analyses demonstrated that the pooled estimates remained materially unchanged following sequential omission of individual studies, comparison of fixed-effects and random-effects models, and exclusion of cohort studies judged to be at very high risk of bias according to the ROBINS-E assessment. These findings confirmed the robustness and consistency of the results (Supplementary Figures S15, S17, S22). Visual inspection of the funnel plots did not reveal obvious asymmetry, and Egger’s tests did not detect statistically significant asymmetry (*p* > 0.05) (Supplementary Figure S19, S21). According to the GRADE assessment, the certainty of evidence was very low for overall atopic diseases and eczema and low for asthma (Table 3).

## 4 Discussion

### 4.1 Interpretation of the overall findings

This study found that prospective cohort studies demonstrated that maternal exposure to n–3 long-chain polyunsaturated fatty acid (LC-PUFA) during pregnancy was significantly associated with a lower risk of overall allergic diseases in offspring. However, randomized controlled trials (RCTs) did not yield statistically significant positive results, which may be attributed to the following reasons: Studies have shown that the anti-inflammatory and immunomodulatory effects of n–3 LC-PUFA exhibit a clear dose-dependent relationship (41, 42). RCTs typically employ standardized fixed-dose intervention protocols, which may not account for baseline supplementation differences among populations and may prevent some individuals from reaching effective exposure levels (5, 17); whereas cohort studies reflect long-term cumulative exposure from dietary intake and routine supplementation in real-world settings, making it easier to reach individual effective thresholds. Additionally, substantial differences in sample size and statistical power may also have influenced the results: In this study, the RCT intervention group included only 2,283 participants, a relatively limited sample size compared with the 63,067 exposed individuals in the cohort studies. This limitation may have reduced the ability to detect potential protective effects.

However, the larger sample size of cohort studies does not eliminate potential confounding. Because the cohort meta-analysis was based on crude event-based RRs, residual confounding cannot be excluded, and differences in maternal characteristics and health behaviors may have contributed to the observed associations. Conversely, although randomization in RCTs generally minimizes confounding, their findings may still be influenced by factors such as intervention dose, adherence, and statistical power. Therefore, the differences between RCT and cohort findings should be interpreted cautiously.

### 4.2 Significant associations with specific allergic outcomes

A prospective cohort study analysis revealed that maternal intake of n–3 long-chain polyunsaturated fatty acids (LC-PUFAs) during pregnancy was significantly associated with a lower risk of asthma (RR = 0.81, 95% CI: 0.68–0.96, *p* = 0.02). This finding was not reported in previous reviews (21), but there was some consistency with certain studies: a dose–response analysis (42) showed that the overall pooled results were not significantly associated with asthma, wheezing, or allergic asthma; however, in European populations, daily supplementation exceeding 1,200 mg and continued from pregnancy through lactation was associated with a lower risk of asthma onset. Another study (43) found that maternal intake of n–3 LC-PUFA during pregnancy was associated with a lower incidence of wheezing/asthma in offspring, but had no significant effect on the overall incidence of childhood asthma.

Additionally, this study also found that maternal intake of n–3 LC-PUFAs during pregnancy was significantly associated with a lower risk of egg allergy in offspring, consistent with previous research (20–22, 44). This finding has important clinical significance, as multiple studies have demonstrated that childhood food allergies, particularly egg allergy, are important predictors of subsequent sensitization to airborne allergens and upper respiratory allergic diseases (45–47), thereby providing a plausible mechanistic explanation for the finding that maternal intake of n–3 LC-PUFAs during pregnancy may be associated with a lower risk of asthma in offspring, providing a potential link between the observed lower risk of egg allergy and subsequent allergic respiratory outcomes, although causality cannot be established by the present analysis.

### 4.3 Insights from subgroup analyses

The results of the subgroup analysis warrant particular attention. In the regional subgroups, both RCTs and prospective cohort studies showed statistically significant associations in European populations, consistent with previous dose–response analyses (42). However, the tests for subgroup differences did not demonstrate statistically significant differences across geographic regions in either RCTs (P for interaction = 0.06) or prospective cohort studies (P for interaction = 0.11). Therefore, the European findings should be interpreted as subgroup-specific associations rather than conclusive evidence of regional effect modification. One plausible explanation is that European populations generally have lower baseline fish and seafood consumption, making it easier for supplementation or higher dietary intake to reach the effective dose threshold required to influence fetal immune programming (42, 48). However, alternative explanations should also be considered, as European studies differed in study design, intervention dosages, follow-up durations, and healthcare settings. Moreover, the cohort findings were based on crude estimates without adjustment for region-specific confounders. Therefore, while this regional finding is hypothesis-generating, it should not be interpreted as conclusive evidence of a population-specific biological effect and warrants confirmation in future studies with harmonized protocols. In Asian populations, positive results were also observed in prospective cohort studies; however, the lack of a statistically significant interaction between regions indicates that these findings should not be interpreted as evidence of a distinct regional effect. The mechanisms underlying regional differences remain unclear. These differences may be related to baseline nutritional status, dietary patterns, and genetic background, and further research is required to elucidate them.

Regarding the timing of intervention initiation, RCT results suggested that interventions initiated at or after 20 weeks of gestation showed more significant effects. This finding may have been influenced by the limited number of included studies and uneven regional distribution. However, the subgroup difference test showed no statistically significant difference (interaction *p* = 0.08), and it is unclear whether the timing of the intervention has an independent effect. Follow-up subgroup analyses showed statistically significant associations during infancy (*p* = 0.03), toddlerhood (*p* = 0.01), and preschool age and above (*p* < 0.001). Importantly, the test for subgroup differences was statistically significant (P for interaction = 0.006), suggesting that the magnitude of the associations differed significantly across follow-up periods. However, this finding should be interpreted cautiously because differences in follow-up duration may also reflect differences in study populations, outcome definitions, and other study characteristics. This finding is partially consistent with Gabryszewski et al., who reported a reduced risk of IgE-mediated allergies in offspring aged 12–36 months but no significant effect before 12 months (28). Similarly, significant differences were observed according to exposure type (P for interaction < 0.001) and disease type (P for interaction = 0.03), indicating that the magnitude of the associations differed across exposure types and allergic outcomes. These findings may reflect differences in the underlying exposure characteristics or outcome definitions, but should be interpreted cautiously given the limited number of studies in some subgroups. However, these subgroup findings should be interpreted cautiously because some subgroups included only a limited number of studies.

In contrast, although a significant protective association was observed among mothers under 30 years old, the subgroup difference test did not reach statistical significance (P for interaction = 0.16), indicating that there was insufficient evidence that maternal age significantly modified the association between prenatal n–3 LC-PUFA intake and allergic outcomes. The observed association might be related to the relatively stable metabolic and physiological state of this population (35–37). However, the underlying mechanism still requires further research to be verified. Overall, the current evidence is insufficient to demonstrate that maternal age modifies the association between prenatal n–3 LC-PUFA intake and allergic outcomes.

### 4.4 Sources of heterogeneity

Regarding the heterogeneity in the study, subgroup analyses demonstrated that variations in follow-up duration (*p* = 0.006), exposure type (*p* < 0.001), and disease type (*p* = 0.03) were the primary sources of heterogeneity.

### 4.5 Strengths and limitations

This meta-analysis has several strengths. It simultaneously includes randomized controlled trials (RCTs) and prospective cohort studies in a comprehensive meta-analysis, systematically comparing the effects of prenatal n–3 LC-PUFA on allergic diseases in offspring under two modes: standardized intervention and natural exposure; Second, this study observed that prenatal exposure to n–3 LC-PUFAs was significantly associated with a lower risk of asthma in offspring (RR = 0.81, 95% CI: 0.68–0.96, *p* = 0.02); Moreover, subgroup analyses were conducted across multiple dimensions, including geographic region and intervention timing, which facilitated the identification of sources of heterogeneity and target populations; Additionally, the included studies featured longer follow-up periods and broader coverage, spanning from infancy to early adulthood, thereby providing a more comprehensive assessment of long-term health outcomes. Furthermore, the certainty of evidence was assessed using the GRADE framework and varied across outcomes and study designs. Among RCTs, the certainty of evidence was low for overall atopic diseases, high for asthma, and moderate for food allergy, whereas the corresponding evidence from prospective cohort studies was rated very low for overall atopic diseases and low for asthma. These differences in certainty should be considered when interpreting the pooled estimates and their clinical implications.

However, this study also has several limitations. First, the number of RCTs and the sample sizes were limited (only eight studies, with 2,283 participants in the intervention group), which may have affected statistical power. Second, some subgroups included a limited number of studies (e.g., the maternal age subgroup with only two studies and the intervention timing subgroup with only four studies), which may have limited statistical power and the stability of the findings. Third, clinical and methodological heterogeneity existed across the included studies, including variations in follow-up duration, intervention dosage and formulation, EPA/DHA composition, and the definitions and assessment methods of allergic outcomes. Such variability may have contributed to outcome misclassification and between-study heterogeneity. Although subgroup analyses partially explored potential sources of heterogeneity, the limited number of studies and incomplete reporting of intervention characteristics prevented reliable dose–response and meta-regression analyses. In addition, although funnel plot inspection and Egger’s tests did not indicate statistically significant asymmetry, the relatively small number of studies (*n* = 8) may have limited the statistical power of Egger’s test. Therefore, publication bias cannot be completely excluded. Fourth, risk-of-bias assessment showed that five of the eight cohort studies were at high or very high risk of bias. In addition, the primary meta-analysis was based on crude event-based RRs, which do not account for potential confounding. This limitation is particularly relevant to prospective cohort studies because maternal n–3 LC-PUFA exposure may be associated with other dietary, lifestyle, socioeconomic, and maternal factors that may also influence allergic disease risk in offspring. Therefore, the observational findings should be interpreted cautiously, and residual confounding cannot be excluded.

Future research should prioritize large-scale, well-designed randomized controlled trials with standardized reporting of n–3 LC-PUFA dosage, EPA/DHA composition, intervention duration, and allergic outcomes to better clarify population-specific and dose-related effects.

## 5 Conclusion

Prenatal n–3 long-chain polyunsaturated fatty acid (n–3 LC-PUFA) intake may be associated with a lower risk of allergic diseases in offspring. The results of prospective cohort studies suggested that maternal n–3 LC-PUFA intake during pregnancy was associated with a lower risk of overall allergic diseases in offspring, with a significant inverse association observed for asthma. The results of randomized controlled trials (RCTs) suggested a potential protective effect in European populations against egg allergy and other related allergic conditions. These findings should be interpreted cautiously, and further high-quality studies are warranted to provide more robust evidence for clinical application and prenatal nutritional guidance.

## Data Availability

The original contributions presented in the study are included in the article/Supplementary material, further inquiries can be directed to the corresponding authors.
