# Supplementary material for: Prenatal n–3 LC-PUFA intake and the incidence of allergic diseases in offspring: an updated systematic review and meta-analysis
Source: Front Nutr. 2026 Sep 24;13:1923649. doi: 10.3389/fnut.2026.1923649 (PMC13645708; doi:10.3389/fnut.2026.1923649)
Supplement: Supplementary file 1 [file Table_1.docx]

Supplementary Material

# Prenatal n–3 LC-PUFA intake and the incidence of Allergic Disease in offspring: An Updated Systematic Review and Meta-Analysis

**Text S1:** Search strategy

**Figure. S1:** Risk of bias graph of randomized controlled trials by each item

**Figure. S2:** Risk of bias summary of randomized controlled trials for each study

**Figure. S3-S13:** Subgroup analysis of outcomes

**Figure. S14-S17:** Sensitivity analysis of outcomes

**Figure. S18-S19:** Funnel plot

**Figure. S20-S21:** Egger's test plot

**Figure. S22:** Sensitivity analysis after excluding studies at Very high risk of bias (ROBINS-E)

**Table S1:** Risk of bias assessment of the prospective cohort study by the ROBINS-E tool

**Table S2.** PRISMA 2020 checklist

# Text S1. Search strategy

**Pubmed**

#1 (Pregnancy[Title/Abstract] OR Pregnan*[Title/Abstract] OR Perinatal[Title/Abstract] OR Prenatal[Title/Abstract] OR Antenatal[Title/Abstract] OR maternal[Title/Abstract] OR Gestation[Title/Abstract])

#2 (Fatty Acids, Omega-3[Title/Abstract] OR N-3 Fatty Acid[Title/Abstract] OR Acid, N-3 Fatty[Title/Abstract] OR Fatty Acid, N-3[Title/Abstract] OR N 3 Fatty Acid[Title/Abstract] OR Omega-3 Fatty Acid[Title/Abstract] OR Acid, Omega-3 Fatty[Title/Abstract] OR Fatty Acid, Omega-3[Title/Abstract] OR Omega 3 Fatty Acid[Title/Abstract] OR Omega-3 Fatty Acids[Title/Abstract] OR n-3 Oil[Title/Abstract] OR n 3 Oil[Title/Abstract] OR Oil, n-3[Title/Abstract] OR n3 Oil[Title/Abstract] OR Oil, n3[Title/Abstract] OR n-3 Fatty Acids[Title/Abstract] OR n 3 Fatty Acids[Title/Abstract] OR n-3 Polyunsaturated Fatty Acid[Title/Abstract] OR n 3 Polyunsaturated Fatty Acid[Title/Abstract] OR n-3 PUFA[Title/Abstract] OR n 3 PUFA[Title/Abstract] OR PUFA, n-3[Title/Abstract] OR n3 Fatty Acid[Title/Abstract] OR Fatty Acid, n3[Title/Abstract] OR n3 PUFA[Title/Abstract] OR PUFA, n3[Title/Abstract] OR n3 Polyunsaturated Fatty Acid[Title/Abstract] OR n3 Oils[Title/Abstract] OR Omega 3 Fatty Acids[Title/Abstract] OR n-3 Oils[Title/Abstract] OR n 3 Oils[Title/Abstract] OR Fish Oils[Title/Abstract] OR Oils, Fish[Title/Abstract] OR Fish Oil[Title/Abstract] OR Oil, Fish[Title/Abstract] OR Fish Liver Oils[Title/Abstract] OR Liver Oils, Fish[Title/Abstract] OR Oils, Fish Liver[Title/Abstract] OR Fatty Acids, Unsaturated[Title/Abstract] OR Acids, Unsaturated Fatty[Title/Abstract] OR Unsaturated Fatty Acids[Title/Abstract] OR Unsaturated Fatty Acid[Title/Abstract] OR Acid, Unsaturated Fatty[Title/Abstract] OR Fatty Acid, Unsaturated[Title/Abstract] OR Polyunsaturated Fatty Acids[Title/Abstract] OR Acids, Polyunsaturated Fatty[Title/Abstract] OR Polyunsaturated Fatty Acid[Title/Abstract] OR Acid, Polyunsaturated Fatty[Title/Abstract] OR Fatty Acid, Polyunsaturated[Title/Abstract] OR Fatty Acids, Polyunsaturated[Title/Abstract] OR PUFA[Title/Abstract] OR PUFAs[Title/Abstract] OR Fatty Acids[Title/Abstract] OR Fatty Acid[Title/Abstract] OR Aliphatic Acids[Title/Abstract] OR Aliphatic Acid[Title/Abstract] OR Acid, Aliphatic[Title/Abstract] OR Fatty Acids, Esterified[Title/Abstract] OR Esterified Fatty Acids[Title/Abstract] OR Esterified Fatty Acid[Title/Abstract] OR Acid, Esterified Fatty[Title/Abstract] OR Fatty Acid, Esterified[Title/Abstract] OR Fatty Acids, Saturated[Title/Abstract] OR Saturated Fatty Acids[Title/Abstract] OR Saturated Fatty Acid[Title/Abstract] OR Acid, Saturated Fatty[Title/Abstract] OR Fatty Acid, Saturated[Title/Abstract] OR Fatty Acids, Essential[Title/Abstract] OR Acids, Essential Fatty[Title/Abstract] OR Essential Fatty Acids[Title/Abstract] OR EFA[Title/Abstract] OR EFAs[Title/Abstract] OR Docosahexaenoic Acids[Title/Abstract] OR Acids, Docosahexaenoic[Title/Abstract] OR Docosahexenoic Acids[Title/Abstract] OR Acids, Docosahexenoic[Title/Abstract] OR Docosahexaenoic Acid[Title/Abstract] OR Acid, Docosahexaenoic[Title/Abstract] OR DHA[Title/Abstract] OR DHAs[Title/Abstract] OR eicosapentenoic acid[Title/Abstract] OR EPA[Title/Abstract] OR Long Chain Triglyceride*[Title/Abstract] OR LCT[Title/Abstract] OR Omegaven[Title/Abstract] OR Smoflipid[Title/Abstract] OR Lipovenoes[Title/Abstract] OR Lipoplus[Title/Abstract] OR Intralipid[Title/Abstract] OR Lipofundin[Title/Abstract] OR Lipoven[Title/Abstract] OR Lipovenoess[Title/Abstract] OR Omegavenous[Title/Abstract] OR Clinoleic[Title/Abstract] OR Structolipid[Title/Abstract] OR Liposyn[Title/Abstract] OR omega 3[Title/Abstract] OR omega 6[Title/Abstract] OR w-3 fatty acid[Title/Abstract] OR n3[Title/Abstract] OR n-3[Title/Abstract] OR n6[Title/Abstract] OR krill oil[Title/Abstract] OR long chain PUFA[Title/Abstract] OR DPA[Title/Abstract] OR epas[Title/Abstract] OR lipid emulsion[Title/Abstract]))

#3 (child[Title/Abstract] OR child*[Title/Abstract] AND offspring[Title/Abstract] OR Infant[Title/Abstract] OR Infan*[Title/Abstract] OR Adolescen*[Title/Abstract] OR Youth*[Title/Abstract]))

#4 (Asthma[Title/Abstract] OR wheez*[Title/Abstract] OR respiratORy*[Title/Abstract] OR IgE-Mediated Hypersensitivit*[Title/Abstract] OR Immediate hypersensitiv*[Title/Abstract] OR Atopic Hypersensitiv*[Title/Abstract] OR Atopy[Title/Abstract] OR Type I Hypersensitiv*[Title/Abstract] OR rhinitis[Title/Abstract] OR Allergic rhinitis[Title/Abstract] OR conjunctivitis[Title/Abstract] OR Allergic conjunctivitis[Title/Abstract] OR Hay fever[Title/Abstract] OR Hayfever[Title/Abstract] OR Atopic Eczema[Title/Abstract] OR Atopic Dermatitis[Title/Abstract] OR Food hypersensitivity[Title/Abstract] OR food allergy[Title/Abstract] OR ORal Allergy Syndrome[Title/Abstract] OR milk allergy[Title/Abstract] OR egg allergy[Title/Abstract] OR nut allergy[Title/Abstract] OR peanut allergy[Title/Abstract] OR arachis hypogaea allergy[Title/Abstract] OR tree nut allergy[Title/Abstract] OR hazelnut allergy[Title/Abstract] OR legumes allergy[Title/Abstract] OR wheat allergy[Title/Abstract] OR soy allergy[Title/Abstract] OR fish allergy[Title/Abstract] OR seafood allergy[Title/Abstract] OR shellfish allergy[Title/Abstract] OR kiwi allergy[Title/Abstract] OR apple allergy[Title/Abstract] OR peach allergy[Title/Abstract] OR additives hypersensitivity[Title/Abstract] OR Allerg*[Title/Abstract])

**Web of Science**

#1 Pregnancy OR Pregnan* OR Perinatal OR Prenatal OR Antenatal OR maternal OR Gestation (Topic)

#2 Fatty Acids, Omega-3 OR N-3 Fatty Acid OR Acid, N-3 Fatty OR Fatty Acid, N-3 OR N 3 Fatty Acid OR Omega-3 Fatty Acid OR Acid, Omega-3 Fatty OR Fatty Acid, Omega-3 OR Omega 3 Fatty Acid OR Omega-3 Fatty Acids OR n-3 Oil OR n 3 Oil OR Oil, n-3 OR n3 Oil OR Oil, n3 OR n-3 Fatty Acids OR n 3 Fatty Acids OR n-3 Polyunsaturated Fatty Acid OR n 3 Polyunsaturated Fatty Acid OR n-3 PUFA OR n 3 PUFA OR PUFA, n-3 OR n3 Fatty Acid OR Fatty Acid, n3 OR n3 PUFA OR PUFA, n3 OR n3 Polyunsaturated Fatty Acid OR n3 Oils OR Omega 3 Fatty Acids OR n-3 Oils OR n 3 Oils OR Fish Oils OR Oils, Fish OR Fish Oil OR Oil, Fish OR Fish Liver Oils OR Liver Oils, Fish OR Oils, Fish Liver OR Fatty Acids, Unsaturated OR Acids, Unsaturated Fatty OR Unsaturated Fatty Acids OR Unsaturated Fatty Acid OR Acid, Unsaturated Fatty OR Fatty Acid, Unsaturated OR Polyunsaturated Fatty Acids OR Acids, Polyunsaturated Fatty OR Polyunsaturated Fatty Acid OR Acid, Polyunsaturated Fatty OR Fatty Acid, Polyunsaturated OR Fatty Acids, Polyunsaturated OR PUFA OR PUFAs OR Fatty Acids OR Fatty Acid OR Aliphatic Acids OR Aliphatic Acid OR Acid, Aliphatic OR Fatty Acids, Esterified OR Esterified Fatty Acids OR Esterified Fatty Acid OR Acid, Esterified Fatty OR Fatty Acid, Esterified OR Fatty Acids, Saturated OR Saturated Fatty Acids OR Saturated Fatty Acid OR Acid, Saturated Fatty OR Fatty Acid, Saturated OR Fatty Acids, Essential OR Acids, Essential Fatty OR Essential Fatty Acids OR EFA OR EFAs OR Docosahexaenoic Acids OR Acids, Docosahexaenoic OR Docosahexenoic Acids OR Acids, Docosahexenoic OR Docosahexaenoic Acid OR Acid, Docosahexaenoic OR DHA OR DHAs OR eicosapentenoic acid OR EPA OR Long Chain Triglyceride* OR LCT OR Omegaven OR Smoflipid OR Lipovenoes OR Lipoplus OR Intralipid OR Lipofundin OR Lipoven OR Lipovenoess OR Omegavenous OR Clinoleic OR Structolipid OR Liposyn OR omega 3 OR omega 6 OR w-3 fatty acid OR n3 OR n-3 OR n6 OR krill oil OR long chain PUFA OR DPA OR epas OR lipid emulsion (Topic)

#3 (child OR child*) AND offspring OR Infant OR Infan* OR Adolescen* OR Youth*(Topic)

#4 Asthma OR wheez* OR respiratory* OR IgE-Mediated Hypersensitivit* OR Immediate hypersensitiv* OR Atopic Hypersensitiv* OR Atopy OR Type I Hypersensitiv* OR rhinitis OR Allergic rhinitis OR conjunctivitis OR Allergic conjunctivitis OR Hay fever OR Hayfever OR Atopic Eczema OR Atopic Dermatitis OR Food hypersensitivity OR food allergy OR ORal Allergy Syndrome OR milk allergy OR egg allergy OR nut allergy OR peanut allergy OR arachis hypogaea allergy OR tree nut allergy OR hazelnut allergy OR legumes allergy OR wheat allergy OR soy allergy OR fish allergy OR seafood allergy OR shellfish allergy OR kiwi allergy OR apple allergy OR peach allergy OR additives hypersensitivity OR Allerg*(Topic)

**Cochrane**

#1 "Pregnancy" OR "Pregnan" OR "Perinatal" OR "Prenatal" OR "Antenatal" OR "maternal" OR "Gestation."

#2 Fatty Acids, Omega-3 OR N-3 Fatty Acid OR Acid, N-3 Fatty OR Fatty Acid, N-3 OR N 3 Fatty Acid OR Omega-3 Fatty Acid OR Acid, Omega-3 Fatty OR Fatty Acid, Omega-3 OR Omega 3 Fatty Acid OR Omega-3 Fatty Acids OR n-3 Oil OR n 3 Oil OR Oil, n-3 OR n3 Oil OR Oil, n3 OR n-3 Fatty Acids OR n 3 Fatty Acids OR n-3 Polyunsaturated Fatty Acid OR n 3 Polyunsaturated Fatty Acid OR n-3 PUFA OR n 3 PUFA OR PUFA, n-3 OR n3 Fatty Acid OR Fatty Acid, n3 OR n3 PUFA OR PUFA, n3 OR n3 Polyunsaturated Fatty Acid OR n3 Oils OR Omega 3 Fatty Acids OR n-3 Oils OR n 3 Oils OR Fish Oils OR Oils, Fish OR Fish Oil OR Oil, Fish OR Fish Liver Oils OR Liver Oils, Fish OR Oils, Fish Liver OR Fatty Acids, Unsaturated OR Acids, Unsaturated Fatty OR Unsaturated Fatty Acids OR Unsaturated Fatty Acid OR Acid, Unsaturated Fatty OR Fatty Acid, Unsaturated OR Polyunsaturated Fatty Acids OR Acids, Polyunsaturated Fatty OR Polyunsaturated Fatty Acid OR Acid, Polyunsaturated Fatty OR Fatty Acid, Polyunsaturated OR Fatty Acids, Polyunsaturated OR PUFA OR PUFAs OR Fatty Acids OR Fatty Acid OR Aliphatic Acids OR Aliphatic Acid OR Acid, Aliphatic OR Fatty Acids, Esterified OR Esterified Fatty Acids OR Esterified Fatty Acid OR Acid, Esterified Fatty OR Fatty Acid, Esterified OR Fatty Acids, Saturated OR Saturated Fatty Acids OR Saturated Fatty Acid OR Acid, Saturated Fatty OR Fatty Acid, Saturated OR Fatty Acids, Essential OR Acids, Essential Fatty OR Essential Fatty Acids OR EFA OR EFAs OR Docosahexaenoic Acids OR Acids, Docosahexaenoic OR Docosahexenoic Acids OR Acids, Docosahexenoic OR Docosahexaenoic Acid OR Acid, Docosahexaenoic OR DHA OR DHAs OR eicosapentenoic acid OR EPA OR Long Chain Triglyceride OR LCT OR Omegaven OR Smoflipid OR Lipovenoes OR Lipoplus OR Intralipid OR Lipofundin OR Lipoven OR Lipovenoess OR Omegavenous OR Clinoleic OR Structolipid OR Liposyn OR omega 3 OR omega 6 OR w-3 fatty acid OR n3 OR n-3 OR n6 OR krill oil OR long chain PUFA OR DPA OR epas OR lipid emulsion

#3 "child" OR "child" OR "offspring" OR "Infant" OR "Infan" OR "Adolescen" OR "Youth."

#4 Asthma OR wheez OR respiratory OR IgE-Mediated Hypersensitivit OR Immediate hypersensitiv OR Atopic Hypersensitiv OR Atopy OR Type I Hypersensitiv OR rhinitis OR Allergic rhinitis OR conjunctivitis OR Allergic conjunctivitis OR Hay fever OR Hayfever OR Atopic Eczema OR Atopic Dermatitis OR Food hypersensitivity OR food allergy OR ORal Allergy Syndrome OR milk allergy OR egg allergy OR nut allergy OR peanut allergy OR arachis hypogaea allergy OR tree nut allergy OR hazelnut allergy OR legumes allergy OR wheat allergy OR soy allergy OR fish allergy OR seafood allergy OR shellfish allergy OR kiwi allergy OR apple allergy OR peach allergy OR additives hypersensitivity OR Allerg

**Medline**

#1 "Pregnancy" OR "Pregnan" OR "Perinatal" OR "Prenatal" OR "Antenatal" OR "maternal" OR "Gestation."

#2 Fatty Acids, Omega-3 OR N-3 Fatty Acid OR Acid, N-3 Fatty OR Fatty Acid, N-3 OR N 3 Fatty Acid OR Omega-3 Fatty Acid OR Acid, Omega-3 Fatty OR Fatty Acid, Omega-3 OR Omega 3 Fatty Acid OR Omega-3 Fatty Acids OR n-3 Oil OR n 3 Oil OR Oil, n-3 OR n3 Oil OR Oil, n3 OR n-3 Fatty Acids OR n 3 Fatty Acids OR n-3 Polyunsaturated Fatty Acid OR n 3 Polyunsaturated Fatty Acid OR n-3 PUFA OR n 3 PUFA OR PUFA, n-3 OR n3 Fatty Acid OR Fatty Acid, n3 OR n3 PUFA OR PUFA, n3 OR n3 Polyunsaturated Fatty Acid OR n3 Oils OR Omega 3 Fatty Acids OR n-3 Oils OR n 3 Oils OR Fish Oils OR Oils, Fish OR Fish Oil OR Oil, Fish OR Fish Liver Oils OR Liver Oils, Fish OR Oils, Fish Liver OR Fatty Acids, Unsaturated OR Acids, Unsaturated Fatty OR Unsaturated Fatty Acids OR Unsaturated Fatty Acid OR Acid, Unsaturated Fatty OR Fatty Acid, Unsaturated OR Polyunsaturated Fatty Acids OR Acids, Polyunsaturated Fatty OR Polyunsaturated Fatty Acid OR Acid, Polyunsaturated Fatty OR Fatty Acid, Polyunsaturated OR Fatty Acids, Polyunsaturated OR PUFA OR PUFAs OR Fatty Acids OR Fatty Acid OR Aliphatic Acids OR Aliphatic Acid OR Acid, Aliphatic OR Fatty Acids, Esterified OR Esterified Fatty Acids OR Esterified Fatty Acid OR Acid, Esterified Fatty OR Fatty Acid, Esterified OR Fatty Acids, Saturated OR Saturated Fatty Acids OR Saturated Fatty Acid OR Acid, Saturated Fatty OR Fatty Acid, Saturated OR Fatty Acids, Essential OR Acids, Essential Fatty OR Essential Fatty Acids OR EFA OR EFAs OR Docosahexaenoic Acids OR Acids, Docosahexaenoic OR Docosahexenoic Acids OR Acids, Docosahexenoic OR Docosahexaenoic Acid OR Acid, Docosahexaenoic OR DHA OR DHAs OR eicosapentenoic acid OR EPA OR Long Chain Triglyceride OR LCT OR Omegaven OR Smoflipid OR Lipovenoes OR Lipoplus OR Intralipid OR Lipofundin OR Lipoven OR Lipovenoess OR Omegavenous OR Clinoleic OR Structolipid OR Liposyn OR omega 3 OR omega 6 OR w-3 fatty acid OR n3 OR n-3 OR n6 OR krill oil OR long chain PUFA OR DPA OR epas OR lipid emulsion

#3 "child" OR "child" OR "offspring" OR "Infant" OR "Infan" OR "Adolescen" OR "Youth."

#4 Asthma OR wheez OR respiratory OR IgE-Mediated Hypersensitivit OR Immediate hypersensitiv OR Atopic Hypersensitiv OR Atopy OR Type I Hypersensitiv OR rhinitis OR Allergic rhinitis OR conjunctivitis OR Allergic conjunctivitis OR Hay fever OR Hayfever OR Atopic Eczema OR Atopic Dermatitis OR Food hypersensitivity OR food allergy OR ORal Allergy Syndrome OR milk allergy OR egg allergy OR nut allergy OR peanut allergy OR arachis hypogaea allergy OR tree nut allergy OR hazelnut allergy OR legumes allergy OR wheat allergy OR soy allergy OR fish allergy OR seafood allergy OR shellfish allergy OR kiwi allergy OR apple allergy OR peach allergy OR additives hypersensitivity OR Allerg

**Embase**

#1 Pregnancy OR Pregnan* OR Perinatal OR Prenatal OR Antenatal OR maternal OR Gestation

#2 Fatty Acids, Omega-3 OR N-3 Fatty Acid OR Acid, N-3 Fatty OR Fatty Acid, N-3 OR N 3 Fatty Acid OR Omega-3 Fatty Acid OR Acid, Omega-3 Fatty OR Fatty Acid, Omega-3 OR Omega 3 Fatty Acid OR Omega-3 Fatty Acids OR n-3 Oil OR n 3 Oil OR Oil, n-3 OR n3 Oil OR Oil, n3 OR n-3 Fatty Acids OR n 3 Fatty Acids OR n-3 Polyunsaturated Fatty Acid OR n 3 Polyunsaturated Fatty Acid OR n-3 PUFA OR n 3 PUFA OR PUFA, n-3 OR n3 Fatty Acid OR Fatty Acid, n3 OR n3 PUFA OR PUFA, n3 OR n3 Polyunsaturated Fatty Acid OR n3 Oils OR Omega 3 Fatty Acids OR n-3 Oils OR n 3 Oils OR Fish Oils OR Oils, Fish OR Fish Oil OR Oil, Fish OR Fish Liver Oils OR Liver Oils, Fish OR Oils, Fish Liver OR Fatty Acids, Unsaturated OR Acids, Unsaturated Fatty OR Unsaturated Fatty Acids OR Unsaturated Fatty Acid OR Acid, Unsaturated Fatty OR Fatty Acid, Unsaturated OR Polyunsaturated Fatty Acids OR Acids, Polyunsaturated Fatty OR Polyunsaturated Fatty Acid OR Acid, Polyunsaturated Fatty OR Fatty Acid, Polyunsaturated OR Fatty Acids, Polyunsaturated OR PUFA OR PUFAs OR Fatty Acids OR Fatty Acid OR Aliphatic Acids OR Aliphatic Acid OR Acid, Aliphatic OR Fatty Acids, Esterified OR Esterified Fatty Acids OR Esterified Fatty Acid OR Acid, Esterified Fatty OR Fatty Acid, Esterified OR Fatty Acids, Saturated OR Saturated Fatty Acids OR Saturated Fatty Acid OR Acid, Saturated Fatty OR Fatty Acid, Saturated OR Fatty Acids, Essential OR Acids, Essential Fatty OR Essential Fatty Acids OR EFA OR EFAs OR Docosahexaenoic Acids OR Acids, Docosahexaenoic OR Docosahexenoic Acids OR Acids, Docosahexenoic OR Docosahexaenoic Acid OR Acid, Docosahexaenoic OR DHA OR DHAs OR eicosapentenoic acid OR EPA OR Long Chain Triglyceride* OR LCT OR Omegaven OR Smoflipid OR Lipovenoes OR Lipoplus OR Intralipid OR Lipofundin OR Lipoven OR Lipovenoess OR Omegavenous OR Clinoleic OR Structolipid OR Liposyn OR omega 3 OR omega 6 OR w-3 fatty acid OR n3 OR n-3 OR n6 OR krill oil OR long chain PUFA OR DPA OR epas OR lipid emulsion

#3 child OR child* AND offspring OR Infant OR Infan* OR Adolescen* OR Youth*

#4 Asthma OR wheez* OR respiratory* OR IgE-Mediated Hypersensitivit* OR Immediate hypersensitiv* OR Atopic Hypersensitiv* OR Atopy OR Type I Hypersensitiv* OR rhinitis OR Allergic rhinitis OR conjunctivitis OR Allergic conjunctivitis OR Hay fever OR Hayfever OR Atopic Eczema OR Atopic Dermatitis OR Food hypersensitivity OR food allergy OR ORal Allergy Syndrome OR milk allergy OR egg allergy OR nut allergy OR peanut allergy OR arachis hypogaea allergy OR tree nut allergy OR hazelnut allergy OR legumes allergy OR wheat allergy OR soy allergy OR fish allergy OR seafood allergy OR shellfish allergy OR kiwi allergy OR apple allergy OR peach allergy OR additives hypersensitivity OR Allerg*

**CINAHL**

#1 Pregnancy OR Pregnan* OR Perinatal OR Prenatal OR Antenatal OR maternal OR Gestation

#2 Fatty Acids, Omega-3 OR N-3 Fatty Acid OR Acid, N-3 Fatty OR Fatty Acid, N-3 OR N 3 Fatty Acid OR Omega-3 Fatty Acid OR Acid, Omega-3 Fatty OR Fatty Acid, Omega-3 OR Omega 3 Fatty Acid OR Omega-3 Fatty Acids OR n-3 Oil OR n 3 Oil OR Oil, n-3 OR n3 Oil OR Oil, n3 OR n-3 Fatty Acids OR n 3 Fatty Acids OR n-3 Polyunsaturated Fatty Acid OR n 3 Polyunsaturated Fatty Acid OR n-3 PUFA OR n 3 PUFA OR PUFA, n-3 OR n3 Fatty Acid OR Fatty Acid, n3 OR n3 PUFA OR PUFA, n3 OR n3 Polyunsaturated Fatty Acid OR n3 Oils OR Omega 3 Fatty Acids OR n-3 Oils OR n 3 Oils OR Fish Oils OR Oils, Fish OR Fish Oil OR Oil, Fish OR Fish Liver Oils OR Liver Oils, Fish OR Oils, Fish Liver OR Fatty Acids, Unsaturated OR Acids, Unsaturated Fatty OR Unsaturated Fatty Acids OR Unsaturated Fatty Acid OR Acid, Unsaturated Fatty OR Fatty Acid, Unsaturated OR Polyunsaturated Fatty Acids OR Acids, Polyunsaturated Fatty OR Polyunsaturated Fatty Acid OR Acid, Polyunsaturated Fatty OR Fatty Acid, Polyunsaturated OR Fatty Acids, Polyunsaturated OR PUFA OR PUFAs OR Fatty Acids OR Fatty Acid OR Aliphatic Acids OR Aliphatic Acid OR Acid, Aliphatic OR Fatty Acids, Esterified OR Esterified Fatty Acids OR Esterified Fatty Acid OR Acid, Esterified Fatty OR Fatty Acid, Esterified OR Fatty Acids, Saturated OR Saturated Fatty Acids OR Saturated Fatty Acid OR Acid, Saturated Fatty OR Fatty Acid, Saturated OR Fatty Acids, Essential OR Acids, Essential Fatty OR Essential Fatty Acids OR EFA OR EFAs OR Docosahexaenoic Acids OR Acids, Docosahexaenoic OR Docosahexenoic Acids OR Acids, Docosahexenoic OR Docosahexaenoic Acid OR Acid, Docosahexaenoic OR DHA OR DHAs OR eicosapentenoic acid OR EPA OR Long Chain Triglyceride* OR LCT OR Omegaven OR Smoflipid OR Lipovenoes OR Lipoplus OR Intralipid OR Lipofundin OR Lipoven OR Lipovenoess OR Omegavenous OR Clinoleic OR Structolipid OR Liposyn OR omega 3 OR omega 6 OR w-3 fatty acid OR n3 OR n-3 OR n6 OR krill oil OR long chain PUFA OR DPA OR epas OR lipid emulsion
#3 child OR child* AND offspring OR Infant OR Infan* OR Adolescen* OR Youth*

#4 Asthma OR wheez* OR respiratory* OR IgE-Mediated Hypersensitivit* OR Immediate hypersensitiv* OR Atopic Hypersensitiv* OR Atopy OR Type I Hypersensitiv* OR rhinitis OR Allergic rhinitis OR conjunctivitis OR Allergic conjunctivitis OR Hay fever OR Hayfever OR Atopic Eczema OR Atopic Dermatitis OR Food hypersensitivity OR food allergy OR ORal Allergy Syndrome OR milk allergy OR egg allergy OR nut allergy OR peanut allergy OR arachis hypogaea allergy OR tree nut allergy OR hazelnut allergy OR legumes allergy OR wheat allergy OR soy allergy OR fish allergy OR seafood allergy OR shellfish allergy OR kiwi allergy OR apple allergy OR peach allergy OR additives hypersensitivity OR Allerg*

**SCOPUS**

#1 "Pregnancy" OR "Pregnan" OR "Perinatal" OR "Prenatal" OR "Antenatal" OR "maternal" OR "Gestation."

#2 Fatty Acids, Omega-3 OR Omega 3 Fatty Acid OR n-3 PUFA OR n-3 Fatty Acid OR n-3 Polyunsaturated Fatty Acid OR Docosahexaenoic Acid OR fish

#3 "child" OR "child" OR "offspring" OR "Infant" OR "Infan" OR "Adolescen" OR "Youth."

#4 Asthma OR "wheez" OR "respiratory" OR "IgE-Mediated Hypersensitivit" OR "Immediate hypersensitiv" OR "Atopic Hypersensitivit" OR "Atopy" OR "Type I Hypersensitiv" OR "Allergic rhinitis" OR "Hay fever" OR "Hayfever" OR "Atopic Eczema" OR "Atopic Dermatitis" OR "Allerg"

**CNKI**

#1 (篇关摘:孕妇 + 产前 +妊娠期 +围产期(模糊))

#2 (篇关摘:n-3脂肪酸+ Omega-3脂肪酸 + w-3脂肪酸 + n-3多不饱和脂肪酸+ Omega-3多不饱和脂肪酸 + w-3多不饱和脂肪酸 + n-3 长链多不饱和脂肪酸 + n-3脂肪酸+Omega-3脂肪酸(模糊))OR(篇关摘:鱼油 + 二十二碳六烯酸 + 二十碳五烯酸 +二十二碳五烯酸 + 磷虾油(模糊))

#3 (篇关摘:后代 + 儿童+婴幼儿+婴儿+ 新生儿 + 青少年 + 子代 + 幼儿(模糊))

#4 (篇关摘:过敏 + 哮喘+湿疹 + 鼻炎 + 特应性 +食物过敏(模糊))

**WanFang**

#1 题名或关键词:(孕期 OR 妊娠 OR 产前 OR 围产期 OR maternal OR 妊娠期 OR 孕中晚期 OR 孕早期 OR 孕妇)

#2 题名或关键词:(n\-3脂肪酸 OR Omega\-3脂肪酸 OR ω\-3脂肪酸 OR n\-3多不饱和脂肪酸 OR Omega\-3多不饱和脂肪酸 OR ω\-3多不饱和脂肪酸 OR n\-3 长链多不饱和脂肪酸) or 题名或关键词:(n\-3脂肪酸 OR Omega\-3脂肪酸 OR ω\-3脂肪酸 OR n\-3多不饱和脂肪酸 OR Omega\-3多不饱和脂肪酸 OR ω\-3多不饱和脂肪酸 OR n\-3 长链多不饱和脂肪酸)

#3 题名或关键词:(后代 OR 儿童 OR 婴幼儿 OR 婴儿 OR 新生儿 OR 青少年 OR 子代 OR 幼儿)

#4 题名或关键词:(过敏性疾病 OR 哮喘 OR 喘息 OR 过敏性鼻炎 OR 变应性鼻炎 OR 过敏性结膜炎 OR 变应性结膜炎 OR 特应性皮炎 OR 特应性湿疹 OR 湿疹) and 题名或关键词:(食物过敏 OR IgE 介导过敏 OR Ⅰ型超敏反应 OR 特应性 OR 过敏体质 OR 皮肤点刺试验阳性 OR 血清IgE升高 OR 花粉症 OR 过敏)

**VIP Database**

#1 ((((((题名或关键词=妊娠期 OR 题名或关键词=产前) OR 题名或关键词=围产期)

#2 ((((题名或关键词=n 3多不饱和脂肪酸)) OR (题名或关键词=Omega 3多不饱和脂肪酸))) OR (题名或关键词=ω 3多不饱和脂肪酸))) OR (题名或关键词=n 3 长链多不饱和脂肪酸)))) OR ((((题名或关键词=鱼油 OR 题名或关键词=二十二碳六烯酸) OR 题名或关键词=二十碳五烯酸) OR 题名或关键词=二十二碳五烯酸) OR 题名或关键词=磷虾油)) AND (((((题名或关键词=后代 OR 题名或关键词=儿童) OR 题名或关键词=婴幼儿) OR 题名或关键词=婴儿) OR 题名或关键词=新生儿) OR 题名或关键词=青少年)) AND (((((((题名或关键词=过敏性疾病 OR 题名或关键词=过敏) OR 题名或关键词=哮喘) OR 题名或关键词=喘息) OR 题名或关键词=鼻炎) OR 题名或关键词=结膜炎) OR 题名或关键词=湿疹) OR 题名或关键词=食物过敏))

**SinoMed**

#1 ( "孕期"[常用字段:智能] OR "妊娠"[常用字段:智能] OR "产前"[常用字段:智能] OR "围产期"[常用字段:智能] OR " maternal"[常用字段:智能] OR "妊娠期"[常用字段:智能] OR "孕中晚期"[常用字段:智能] OR "孕早期"[常用字段:智能] OR "孕妇"[常用字段:智能])

#2 ( "2.n-3脂肪酸"[常用字段:智能] OR "Omega-3脂肪酸"[常用字段:智能] OR "ω-3脂肪酸"[常用字段:智能] OR "n-3多不饱和脂肪酸"[常用字段:智能] OR "Omega-3多不饱和脂肪酸"[常用字段:智能] OR "ω-3多不饱和脂肪酸"[常用字段:智能] OR "n-3 长链多不饱和脂肪酸"[常用字段:智能] OR "鱼油"[常用字段:智能] OR "DHA"[常用字段:智能] OR "二十二碳六烯酸"[常用字段:智能] OR "EPA"[常用字段:智能] OR "二十碳五烯酸"[常用字段:智能] OR "n-3 PUFA"[常用字段:智能] OR "PUFA"[常用字段:智能] OR "多不饱和脂肪酸"[常用字段:智能] OR "深海鱼油"[常用字段:智能] OR "鱼油胶囊"[常用字段:智能] OR "n-3脂肪酸补充剂"[常用字段:智能] OR "Omega-3补充剂"[常用字段:智能] OR "长链多不饱和脂肪酸（LC-PUFA）"[常用字段:智能] OR "二十二碳五烯酸"[常用字段:智能] OR "DPA"[常用字段:智能] OR "磷虾油"[常用字段:智能] OR "n3 脂肪酸"[常用字段:智能])

#3 ( "3.后代"[常用字段:智能] OR "儿童"[常用字段:智能] OR "婴幼儿"[常用字段:智能] OR "婴儿"[常用字段:智能] OR "新生儿"[常用字段:智能] OR "青少年"[常用字段:智能] OR "子代"[常用字段:智能] OR "幼儿"[常用字段:智能])

#4 ( "4.过敏性疾病"[常用字段:智能] OR "哮喘"[常用字段:智能] OR "喘息"[常用字段:智能] OR "过敏性鼻炎"[常用字段:智能] OR "变应性鼻炎"[常用字段:智能] OR "过敏性结膜炎"[常用字段:智能] OR "变应性结膜炎"[常用字段:智能] OR "特应性皮炎"[常用字段:智能] OR "特应性湿疹"[常用字段:智能] OR "湿疹"[常用字段:智能] OR "食物过敏"[常用字段:智能] OR "IgE 介导过敏"[常用字段:智能] OR "Ⅰ型超敏反应"[常用字段:智能] OR "特应性"[常用字段:智能] OR "过敏体质"[常用字段:智能] OR "皮肤点刺试验阳性"[常用字段:智能] OR "血清IgE升高"[常用字段:智能] OR "花粉症"[常用字段:智能] OR "鸡蛋过敏"[常用字段:智能] OR "牛奶过敏"[常用字段:智能] OR "坚果过敏"[常用字段:智能] OR "花生过敏"[常用字段:智能] OR "海鲜过敏"[常用字段:智能] OR "过敏性喘息"[常用字段:智能] OR "持续性喘息"[常用字段:智能] OR "过敏性皮肤病"[常用字段:智能])

**Figure S1.** Risk of bias graph of randomized controlled trials by each item

**Figure S2.** Risk of bias summary of randomized controlled trials for each study

**Figure S3.** Forest plot of subgroup analysis by region (RCTs)

**Figure S4.** Forest plot of subgroup analysis by EPA-to-DHA ratio (RCTs)

**Figure S5.** Forest plot of subgroup analysis by follow-up duration (RCTs)

**Figure S6.** Forest plot of subgroup analysis by intervention initiation time (RCTs)

**Figure S7.** Forest plot of subgroup analysis by types of allergic disease (RCTs)

**Figure S8.** Forest plot of subgroup analysis by types of food allergies (RCTs)

**Figure S9.** Forest plot of subgroup analysis by region (prospective cohort studies)

**Figure S10.** Forest plot of subgroup analysis by follow-up duration (prospective cohort studies)

**Figure S11.** Forest plot of subgroup analysis by exposure type (prospective cohort studies)

**Figure S12.** Forest plot of subgroup analysis by maternal age (prospective cohort studies)

**Figure S13.** Forest plot of subgroup analysis by type of allergic disease (prospective cohort studies)

**Figure S14.** Sensitivity analysis of the randomized controlled trial study

**Figure S15.** Sensitivity analysis of the prospective cohort study

**Figure S16.** Conduct a sensitivity analysis for the randomized controlled trial study using the fixed effect model

**Figure S17.** Conduct a sensitivity analysis for the prospective cohort study using the fixed effect model

**Figure S18.** Funnel plot of the randomized controlled trial study

**Figure S19.** Funnel plot of the prospective cohort study

**Figure S20.** Egger's test plot of the randomized controlled trial study

**Figure S21.** Egger's test plot of the prospective cohort study

**Figure S22.** Sensitivity analysis after excluding studies at Very high risk of bias (R

OBINS-E)

**Table S1.** Risk of bias assessment of the prospective cohort study by the ROBINS-E tool

| First Author (Year) | D1 (Confounding) | D2 (Classification of Exposure) | D3  (Selection of Participants) | D4  (Deviation from Intended Interventions) | D5  (Missing Data) | D6  (Outcome Measurement) | D7  (Selective Reporting) | Overall Risk of Bias |
| --- | --- | --- | --- | --- | --- | --- | --- | --- |
| Chatzi 2008 [13] | Some concerns | Low risk | Some concerns | Low risk | Some concerns | Low risk | Some concerns | Some concerns |
| Miyake 2009 [34] | Some concerns | Low risk | High risk | Low risk | High risk | Low risk | Some concerns | High risk |
| Maslova 2013 [18] | Low risk | Low risk | High risk | Low risk | Low risk | Low risk | Some concerns | High risk |
| Miyake 2013 [35] | Some concerns | Low risk | High risk | Low risk | Some concerns | Low risk | Some concerns | High risk |
| Sausenthaler 2007 [36] | Some concerns | Very high risk | Some concerns | Low risk | Very high risk | Low risk | Some concerns | Very high risk |
| Henry 2025 [33] | Some concerns | Low risk | Some concerns | Low risk | Some concerns | Low risk | Low risk | Some concerns |
| Tsuji 2024 [15] | Low risk | Low risk | Some concerns | Low risk | Low risk | Low risk | Some concerns | Some concerns |
| Jedrychowski 2011 [32] | Some concerns | Low risk | High risk | Low risk | Some concerns | Low risk | Low risk | High risk |

D1 (Confounding): Assesses whether important confounding factors were appropriately accounted for in the analysis.

D2 (Classification of Exposure): Assesses whether the exposure was measured or classified accurately and whether measurement error or misclassification could have introduced bias.

D3 (Selection of Participants): Assesses whether selection of participants into the study or analysis could have introduced bias.

D4 (Deviation from Intended Interventions): Assesses whether interventions or changes occurring after exposure measurement could have introduced bias.

D5 (Missing Data): Assesses whether missing data could have biased the estimated effect and whether missing data were appropriately handled.

D6 (Outcome Measurement): Assesses whether the outcome was measured accurately and consistently and whether outcome measurement could have introduced bias.

D7 (Selective Reporting): Assesses whether selection of the reported result from multiple eligible analyses or outcomes could have introduced bias.

Overall risk of bias: The overall risk of bias was categorized as “Low risk,” “Some concerns,” “High risk,” or “Very high risk.”

**Table S2.** PRISMA 2020 checklist

| **Section and Topic** | **Item #** | **Checklist item** | **Location where item is reported** |
| --- | --- | --- | --- |
| **TITLE** | | |  |
| Title | 1 | Identify the report as a systematic review. | Page 1 |
| **ABSTRACT** | | |  |
| **INTRODUCTION** | | |  |
| Rationale | 3 | Describe the rationale for the review in the context of existing knowledge. | Page 2-3 |
| Objectives | 4 | Provide an explicit statement of the objective(s) or question(s) the review addresses. | Page 2-3 |
| **METHODS** | | |  |
| Eligibility criteria | 5 | Specify the inclusion and exclusion criteria for the review and how studies were grouped for the syntheses. | Page 3 |
| Information sources | 6 | Specify all databases, registers, websites, organisations, reference lists and other sources searched or consulted to identify studies. Specify the date when each source was last searched or consulted. | Page 3 |
| Search strategy | 7 | Present the full search strategies for all databases, registers and websites, including any filters and limits used. | Page 2-18 of the supplementary materials |
| Selection process | 8 | Specify the methods used to decide whether a study met the inclusion criteria of the review, including how many reviewers screened each record and each report retrieved, whether they worked independently, and if applicable, details of automation tools used in the process. | Page 3-4 |
| Data collection process | 9 | Specify the methods used to collect data from reports, including how many reviewers collected data from each report, whether they worked independently, any processes for obtaining or confirming data from study investigators, and if applicable, details of automation tools used in the process. | Page 3-4 |
| Data items | 10a | List and define all outcomes for which data were sought. Specify whether all results that were compatible with each outcome domain in each study were sought (e.g. for all measures, time points, analyses), and if not, the methods used to decide which results to collect. | Page 3-5 |
|  | 10b | List and define all other variables for which data were sought (e.g. participant and intervention characteristics, funding sources). Describe any assumptions made about any missing or unclear information. | Page 3-5 |
| Study risk of bias assessment | 11 | Specify the methods used to assess risk of bias in the included studies, including details of the tool(s) used, how many reviewers assessed each study and whether they worked independently, and if applicable, details of automation tools used in the process. | Page 4 |
| Effect measures | 12 | Specify for each outcome the effect measure(s) (e.g. risk ratio, mean difference) used in the synthesis or presentation of results. | Page 3-5 |
| Synthesis methods | 13a | Describe the processes used to decide which studies were eligible for each synthesis (e.g. tabulating the study intervention characteristics and comparing against the planned groups for each synthesis (item #5)). | Page 4 |
|  | 13b | Describe any methods required to prepare the data for presentation or synthesis, such as handling of missing summary statistics, or data conversions. | Page 4 |
|  | 13c | Describe any methods used to tabulate or visually display results of individual studies and syntheses. | Page 3-4 |
|  | 13d | Describe any methods used to synthesize results and provide a rationale for the choice(s). If meta-analysis was performed, describe the model(s), method(s) to identify the presence and extent of statistical heterogeneity, and software package(s) used. | Page 4-5 |
|  | 13e | Describe any methods used to explore possible causes of heterogeneity among study results (e.g. subgroup analysis, meta-regression). | Page 4-6 |
|  | 13f | Describe any sensitivity analyses conducted to assess robustness of the synthesized results. | Page 4 |
| Reporting bias assessment | 14 | Describe any methods used to assess risk of bias due to missing results in a synthesis (arising from reporting biases). | Not reported |
| Certainty assessment | 15 | Describe any methods used to assess certainty (or confidence) in the body of evidence for an outcome. | Page 4 |
| **RESULTS** | | |  |
| Study selection | 16a | Describe the results of the search and selection process, from the number of records identified in the search to the number of studies included in the review, ideally using a flow diagram. | Page 6 |
|  | 16b | Cite studies that might appear to meet the inclusion criteria, but which were excluded, and explain why they were excluded. | Figure 1 |
| Study characteristics | 17 | Cite each included study and present its characteristics. | Page 15-16 |
| Risk of bias in studies | 18 | Present assessments of risk of bias for each included study. | Page 6-7, 19, Page 25-26 of the supplementary materials |
| Results of individual studies | 19 | For all outcomes, present, for each study: (a) summary statistics for each group (where appropriate) and (b) an effect estimate and its precision (e.g. confidence/credible interval), ideally using structured tables or plots. | Figure 2,3 |
| Results of syntheses | 20a | For each synthesis, briefly summarise the characteristics and risk of bias among contributing studies. | Page 6-7 |
|  | 20b | Present results of all statistical syntheses conducted. If meta-analysis was done, present for each the summary estimate and its precision (e.g. confidence/credible interval) and measures of statistical heterogeneity. If comparing groups, describe the direction of the effect. | Page 6-7 |
|  | 20c | Present results of all investigations of possible causes of heterogeneity among study results. | Page 6-7 |
|  | 20d | Present results of all sensitivity analyses conducted to assess the robustness of the synthesized results. | Page 6-7, Page 20-21, 24 of the supplementary materials |
| Reporting biases | 21 | Present assessments of risk of bias due to missing results (arising from reporting biases) for each synthesis assessed. | Page 6-7, Page 22-23 of the supplementary materials |
| Certainty of evidence | 22 | Present assessments of certainty (or confidence) in the body of evidence for each outcome assessed. | Page 6-7 |
| **DISCUSSION** | | |  |
| Discussion | 23a | Provide a general interpretation of the results in the context of other evidence. | Page 7-8 |
|  | 23b | Discuss any limitations of the evidence included in the review. | Page 10 |
|  | 23c | Discuss any limitations of the review processes used. | Page 10 |
|  | 23d | Discuss implications of the results for practice, policy, and future research. | Page 10 |
| **OTHER INFORMATION** | | |  |
| Registration and protocol | 24a | Provide registration information for the review, including register name and registration number, or state that the review was not registered. | Page 3 |
|  | 24b | Indicate where the review protocol can be accessed, or state that a protocol was not prepared. | Not reported |
|  | 24c | Describe and explain any amendments to information provided at registration or in the protocol. | Not reported |
| Support | 25 | Describe sources of financial or non-financial support for the review, and the role of the funders or sponsors in the review. | Page 11 |
| Competing interests | 26 | Declare any competing interests of review authors. | Page 11 |
| Availability of data, code and other materials | 27 | Report which of the following are publicly available and where they can be found: template data collection forms; data extracted from included studies; data used for all analyses; analytic code; any other materials used in the review. | Page 11 |

1	Chatzi, L., M. Torrent, I. Romieu, R. Garcia-Esteban, et al., *Mediterranean diet in pregnancy is protective for wheeze and atopy in childhood.* Thorax, 2008. **63**(6): 507–513.

2	Miyake, Y., S. Sasaki, K. Tanaka, S. Ohfuji, et al., *Maternal fat consumption during pregnancy and risk of wheeze and eczema in Japanese infants aged 16-24 months: the Osaka Maternal and Child Health Study.* Thorax, 2009. **64**(9): 815–821.

3	Maslova, E., M. Strom, E. Oken, H. Campos, et al., *Fish intake during pregnancy and the risk of child asthma and allergic rhinitis - longitudinal evidence from the Danish National Birth Cohort.* British Journal of Nutrition, 2013. **110**(7): 1313–1325.

4	Miyake, Y., K. Tanaka, H. Okubo, S. Sasaki, et al., *Maternal fat intake during pregnancy and wheeze and eczema in Japanese infants: the Kyushu Okinawa Maternal and Child Health Study.* Ann Epidemiol, 2013. **23**(11): 674–680.

5	Sausenthaler, S., S. Koletzko, B. Schaaf, I. Lehmann, et al., *Maternal diet during pregnancy in relation to eczema and allergic sensitization in the offspring at 2 y of age.* American Journal of Clinical Nutrition, 2007. **85**(2): 530–537.

6	Henry, C.O., P.J. Allsopp, A.J. Yeates, T. Spence, et al., *Associations between maternal fish intake and polyunsaturated fatty acid status with childhood asthma in a high fish-eating population.* Pediatr Allergy Immunol, 2025. **36**(1): e70019.

7	Tsuji, S., Y. Adachi, A. Tsuchida, K. Hamazaki, et al., *Association of allergies in children younger than 3 years with levels of maternal intake of n-3 polyunsaturated fatty acids or fish during pregnancy: A nationwide birth cohort study, the Japan Environment and Children's Study.* Allergology International, 2024. **73**(2): 282–289.

8	Jedrychowski, W., F. Perera, U. Maugeri, D. Mrozek-Budzyn, et al., *Effects of Prenatal and Perinatal Exposure to Fine Air Pollutants and Maternal Fish Consumption on the Occurrence of Infantile Eczema.* International Archives of Allergy and Immunology, 2011. **155**(3): 275–281.
